# SSH-DAuth: secret sharing based decentralized OAuth using decentralized identifier

**DOI:** 10.1038/s41598-023-44586-6

**Published:** 2023-10-26

**Authors:** Danda Prudhvi Krishna, R. Ramaguru, K. Praveen, M. Sethumadhavan, Kattur Soundarapandian Ravichandran, Raghunathan Krishankumar, Amir H. Gandomi

**Affiliations:** 1 TIFAC-CORE in Cyber Security, Amrita School of Engineering, Amrita Vishwa Vidyapeetham, Coimbatore, India; 2 Department of Mathematics, School of Physical Sciences, Amrita Vishwa Vidyapeetham, Coimbatore, India; 3 Information Technology Systems and Analytics Area, Indian Institute of Management Bodh Gaya, Bodh Gaya, Bihar 824234 India; 4 Faculty of Engineering and Information Technology, University of Technology Sydney, Ultimo, NSW 2007 Australia; 5 University Research and Innovation Center (EKIK), Obuda University, 1034 Budapest, Hungary

**Keywords:** Engineering, Mathematics and computing

## Abstract

OAuth2.0 is a Single Sign-On approach that helps to authorize users to log into multiple applications without re-entering the credentials. Here, the OAuth service provider controls the central repository where data is stored, which may lead to third-party fraud and identity theft. To circumvent this problem, we need a distributed framework to authenticate and authorize the user without third-party involvement. This paper proposes a distributed authentication and authorization framework using a secret-sharing mechanism that comprises a blockchain-based decentralized identifier and a private distributed storage via an interplanetary file system. We implemented our proposed framework in Hyperledger Fabric (permissioned blockchain) and Ethereum TestNet (permissionless blockchain). Our performance analysis indicates that secret sharing-based authentication takes negligible time for generation and a combination of shares for verification. Moreover, security analysis shows that our model is robust, end-to-end secure, and compliant with the Universal Composability Framework.

## Introduction

Authentication and authorization play a crucial part in security frameworks by affirming a client’s identity and allowing access to web applications. Therefore, organizations should deploy robust authentication mechanisms to verify the identity of the end users and prevent data breaches. In 2016, 154 million US voter records were exposed due to data breach^1^. In the same year, the world tech firm Capgemini had a database leak of personal information of potentially millions of users of a global recruiting firm^2^. Generally, many web applications are being developed and used for availing various services; as a result, users are required to remember multiple login credentials, which becomes a monumental task. Various frameworks have been proposed to overcome this, such as Single Sign-On (SSO)^3^ in which end users are validated only once at a trusted platform called the Identity Provider (*IdP*) and afterward login to different Service Providers ($S_{p}$) without re-entering credentials. Thus, SSO simplifies user authentication by remembering the master credentials. Companies like Google, Facebook, and Microsoft have used SSO to authenticate legitimate users.

In the current generation, providing security for communication on the Internet is a complex and challenging task. SSO is susceptible to attacks like XML injection, On-Path Attacks, and Authentication bypass due to improper implementation on the client side. Various methods like privacy-based adaptive SSO^4^ make the system simple for the user and provide authentication security to $S_{p}$ for their applications. Verifiable Encryption SSO^5^, which uses a mathematical algorithm via a one-time pad (OTP) for authentication, was proposed for securing SSO. Security Assertion Markup Language (SAML)^6^ and OpenID Connect^7^ are the most widely implemented protocols in SSO. The OAuth 2.0 framework^8^ provides authentication and authorization using the user’s credentials in an existing centralized identity provider. OpenID Connect builds an identity layer on top of the OAuth 2.0 framework.

### Motivation and objective

There are limitations in the current SSO, and our framework addresses these limitations by leveraging the latest technologies. The motivation and objectives of the proposed work are as follows:

- To improve user identity management by creating a more secure and reliable authentication and authorization system that takes advantage of blockchain technology’s decentralized, & immutable nature.
- To prevent unauthorized access and lessen the danger of data breaches, user identities are distributed and protected utilizing secret sharing.
- To establish a system resilient to single points of failure and impervious to censorship and hacking, the work investigates the use of the Interplanetary File System (IPFS).
- Traditional authentication techniques can be more prone to new dangers as technology develops. We aim to develop secure and privacy-conscious SSO systems, paving the way for more reliable and user-centric identity management solutions across diverse services.

### Our contribution

In this paper, we proposed and implemented a blockchain-enabled distributed authorization scheme designed to perform SSO in the zero-trust environment. We have added a secret-sharing mechanism allowing participants to split the user Decentralized Identifier (DID) into several shares so that each user has a mandatory share of their DID. The DID can only be reconstructed when sufficient shares are combined with a mandatory share for authorization. The following are the main contributions of our framework.

- Using our model, participants can authenticate independently without relying on a Trusted Third Party (TTP).
- We showed that our proposed model is secure based on the universal composability framework and guarantees fairness by authenticating the users through DID and smart contracts.
- To the best of our knowledge, this is the first work proposed on a Distributed Authentication framework based on DID using a secret sharing mechanism.
- There are limitations in OAuth2.0, and our framework addresses these limitations by leveraging the latest technologies. Table 1 compares our proposed model with the OAuth2.0 framework.

**Table 1:** Comparison of OAuth2.0 with SSH-DAuth.

Factors	OAuth2.0	SSH-DAuth
Centralization	Centralized	Decentralized
DID Enabled	Not used	Used
Data confidentiality	Public key infrastructure	Secret Sharing
Data privacy	Not anonymous	Pseudo-anonymous
Availability	Single point of failure	No downtime
**Transaction anonymity**	Not Anonymous	Pseudo-anonymous

## Preliminaries

### Identity access management

Identification of authorized users who can use the appropriate resources within the organization is made through the Identity Access Management (IAM) system^9^. The three types of access management include Independent Identity Management (IIM), Centralized Identity Management (CIM), and Federated Identity Management (FIM). IIM and FIM model supports multiple *IdP*’s whereas CIM System has only one *IdP*. SSO approach, which Google and Microsoft widely adopt, falls under the FIM model. Here in our framework, we are following the FIM model.

### Authentication schemes

This section reviews various authentication schemes in which the users must prove their identity before accessing data.

#### Security assertion markup language (SAML)

**Figure 1:**
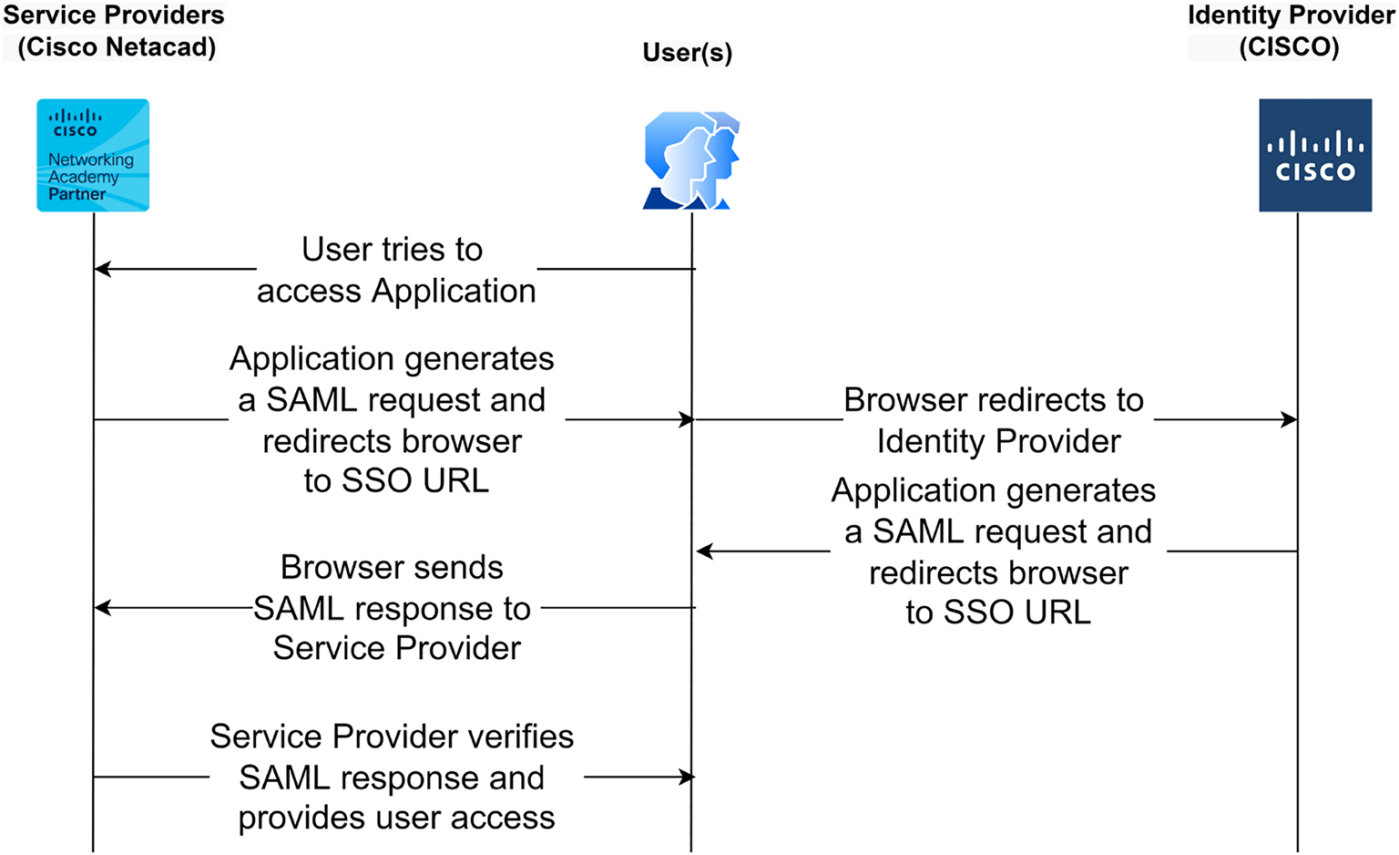
Timeline diagram of SAML.

SAML was developed by the OASIS foundation and was released in March 2005. It is an open standard for authorization and authentication, allowing two web entities to exchange data. SAML assertions are used as security tokens for authenticating the users. As this assertion contains security claims about the subject, the validity of these claims should be certified. This validation can be done using Extensible Markup Language (XML) signatures, which should cover the entire SAML assertion. SAML supports XML, HTTP, SOAP, and other protocols that can transfer XML Signatures. The working of SAML is described through the timeline diagram in Fig. 1.

**Figure 2:**
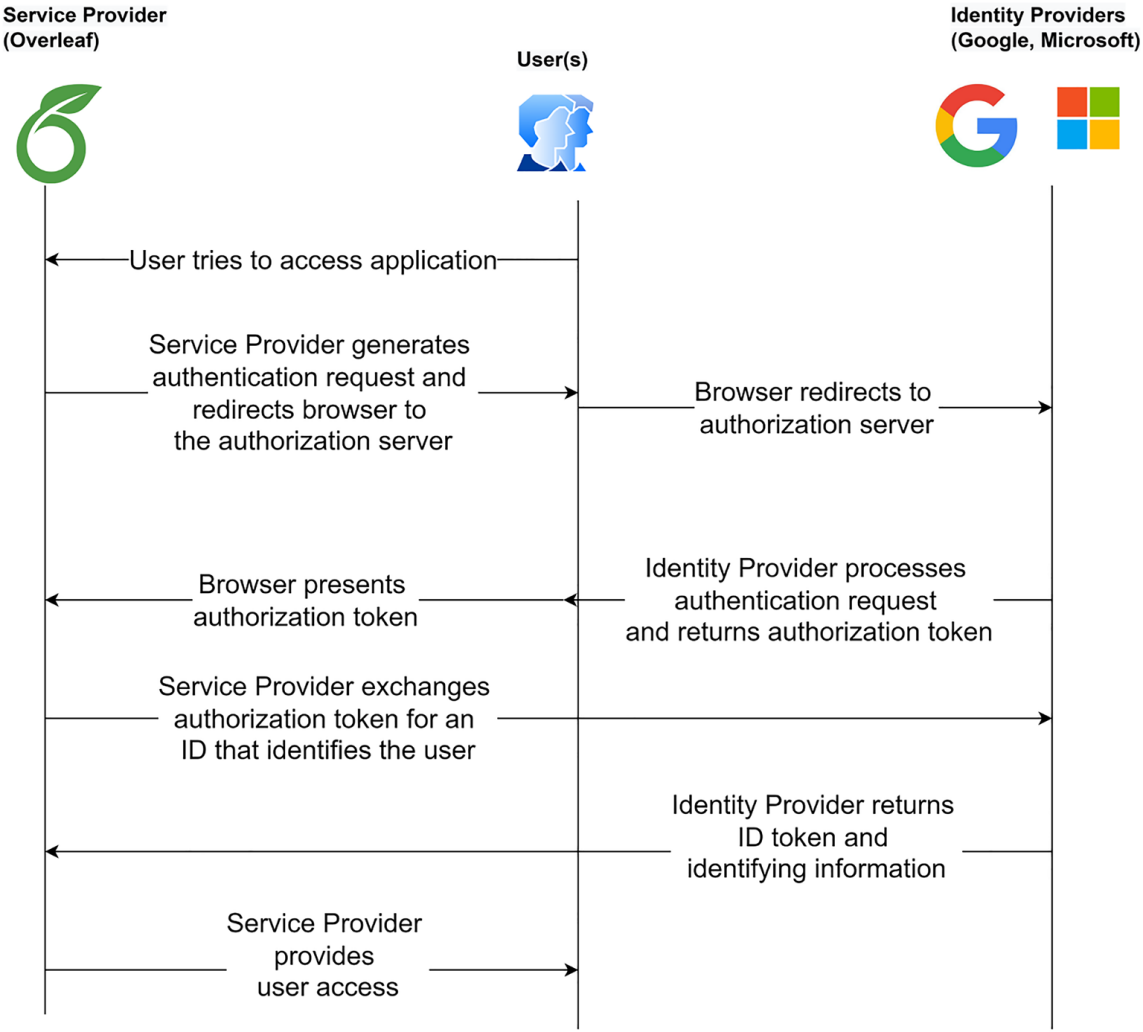
Timeline diagram of OpenID Connect and OAuth.

**Table 2:** SAML, OpenID Connect and OAuth2.0 Specifications.

	SAML	OpenID connect	OAuth2.0
Open Standard for	Authorization and Authentication	Authentication	Authorization
Developed by	OASIS	OpenID Foundation	Twitter and Google
Developed in	2001	2014	2006
Primary usecase is SSO for	Enterprise Apps	Consumer Apps	API Authorization
Used from	2001	2014	2012
When to use	User or corporate partner to access web service	Authenticate users without an account	Temporary resource access to 3rd party apps on a legitimate user’s behalf
Security	XML Signing	Access token validation	Access token validation

#### OpenID connect and OAuth

*OpenID Connect* is an open authentication standard that adds a fundamental identity layer to OAuth. It allows clients to verify the end-user’s identity via authentication performed by an authorization server. OAuth, an authorization standard developed by Twitter and Google, gives brief assets for legitimate clients to get to third-party applications. In expansion, it gives clients designated security to server assets on behalf of an asset owner. The working of the OpenID and OAuth is described through the timeline diagram in Fig. 2. The comparison between SAML, OpenID Connect, and OAuth2.0 has been shown in Table 2.

### Self-sovereign identity model

The Self-Sovereign Identity (SSI) model provides a secure digital identity in which the user controls their information^10^. This model provides a trusted relationship between the user and websites to access the protected resources without relying on any central repository. SSI is made of claims, proofs, and assertions, whereby claims are the identities the user creates when registering with the blockchain. Proofs are documents that act as evidence for the claims, and assertions are stored in the user’s device that the other parties validate to check whether the claims are valid.

**Figure 3:**
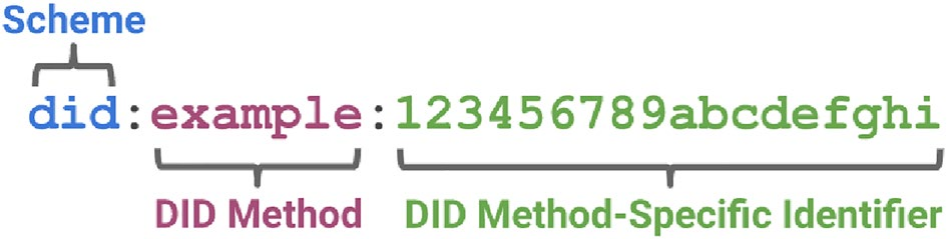
Sample DID. Source:w3.org^11^.

### Decentralized identifiers

A Decentralized Identifier (DID)^12^ is a globally unique and persistent identifier developed as a standard by the World Wide Web Consortium (W3C) as shown in Fig. 3 that offers verifiable and decentralized digital identity. DIDs are essential components of SSI, created and controlled by individual users. A DID maps to a DID document that contains a series of claims about the user’s identity. It is communicated as the linchpin of SSI and employs blockchain or another Distributed Ledger Technology (DLT) to secure privacy and security concerns. It provides faster verification, privacy protection, and selective disclosure of information through the Zero-Knowledge Protocol (ZKP). Each DID has its method, as shown in Table 3

**Table 3:** Active DID Method Specifications.

Method	DID prefix
Sovrin	did:sov:
IPDB	did:ipdb:
Ethereum uPort	did:uport:
IPFS	did:ipld:
Amrita^13^	did:avvcyber:

### Blockchain technology

Blockchain Technology is a decentralized computation and distributed ledger platform that efficiently stores immutable transactions in a verifiable manner through a rational decision-making process among multiple parties in an open and public system^14^. Blockchain allows individuals and companies to instantly store and safely exchange data and value. Information in Blockchain is transferred peer-to-peer without any middlemen or intermediaries. Users have access to see every transaction made on a permissionless blockchain, which is open and transparent. On the other hand, access and visibility are controlled by a permissioned blockchain. Bitcoin, the world’s first cryptocurrency, is an example of a permissionless blockchain employing the Proof of Work (PoW) consensus algorithm. Ripple is a permissioned blockchain network that uses the ledger consensus protocol to verify each transaction.

#### Ethereum

Ethereum^15^ is a permissionless programmable blockchain that stores and executes programs called smart contracts for building decentralized applications (DApps). Ethereum runs on a virtual environment called the Ethereum Virtual Machine (EVM) to isolate itself from the typical ecosystem. Smart contracts are the fundamental building blocks of Ethereum applications. Smart Contracts are self-executing code deployed and executed on a distributed ledger technology when predetermined conditions are met^16, 17^. Smart contracts in Ethereum are written in Solidity language.

Various Ethereum Request for Comments (ERC) standards are available to handle Distributed Identity, which include but are not limited to ERC-1056: Ethereum Lightweight Identity^18^, ERC-1207: DAuth Access Delegation Standard^19^, ERC-1484: Digital Identity Aggregator^20^, and ERC-4361: Sign-In with Ethereum^21^.

#### Hyperledger fabric

Hyperledger Fabric^22^ is an open-source blockchain framework developed under the Hyperledger project. It offers a modular architecture that enables organizations to create permissioned blockchain networks and decentralized apps. Fabric supports programmable logic called chaincode, private channels, and pluggable consensus algorithms. Chaincode^23^ is the business logic deployed on the network to enable users to interact with the blockchain and perform various actions, like reading or modifying the ledger or invoking transactions. Chaincode runs in a secured docker container isolated from the endorsing peer process. Chaincode initializes and manages the ledger state through transactions submitted by applications. Chaincode is written in Go, node.js, or Java that implements a prescribed interface.

### IPFS

Interplanetary File System (IPFS)^24^ is a peer-to-peer network system for storing and accessing data. As a content-addressed protocol, IPFS splits each file into smaller chunks that are hashed cryptographically and are given a unique fingerprint called a Content Identifier (CID).

## Related works

Many researchers have introduced frameworks for implementing secure authentication. For instance, Teja^25^ implemented a safe authentication system for preventing phishing attacks by using secret sharing and QR code scanning. This mechanism works on a dedicated mobile application, which eliminates the process of logging in via user credentials. According to this system, when the user scans the QR code, the mobile application generates the code to an authentication server. The server validates the code using Lagrange’s polynomial and gives access to the user’s protected resources.

Seong-ho Hong^26^ proposed a new SSI-based OAuth model named Vault-point, which provides decentralization and integrity to the user. Vault-point uses the Ethereum platform and consists of three types of smart contracts, namely- Identification contract, Notification contract, and Client management contract. The Identification contract stores the information related to the user, who can edit, delete, and update his identity. The Notification contract delivers the client’s authorization request to the corresponding user’s device. In the Client management contract, the client’s (service provider) information will be stored and executed when the user wants to connect to the service provider.

Nikos Fotiou proposed a token-based OAuth2.0 using distributed ledger^27^. In this token system, the resource server grants permission to the protected user data by validating the ERC-721 token corresponding to the JSON Web Tokens (JWT) received from the client. Anjum^28^ developed a distributed framework for storing patients’ medical records (PMR) based on the Ethereum blockchain. The ERC-721 standard tokenizes these records, which are then stored in the privately distributed storage known as IPFS. Furthermore, to provide complete control over the medical records of the patients, the proposed framework incorporates a Non-Linear Secret Sharing (NLSS) scheme of (1, *t*, *n*).

Soumyashree^29^ designed a blockchain-based distributed IoT architecture for secure authentication and key management. This method specializes in achieving authentication using a one-way hash chain technique, in which cryptographic hash values are generated from a single key that is impossible to revert. This framework includes three layers, namely, device, fog, and cloud layers. The access managing nodes (AMNs) displayed in the fog layer oversee the devices present within the device layer. These AMNs are gathered to create a blockchain network that generates, distributes, and manages the secret keys. The entire transactions are validated and processed by the AMNs between the layers.

Hadjer Benhadj^30^ introduced a lightweight blockchain-based verification mechanism to eliminate the single point failure and reduce the communication overhead and validation from the centralized Public-key Infrastructure (PKI). The strategy addresses these issues by including decentralized blockchain validators’ admission/revocation details. As a result, no IoT device should add its certificate to each message, as the blockchain network will validate its entry.

Shibasis Patel^31^ proposed an authentication service based on the Ethereum blockchain called DAuth, in which the user’s session will be activated by validating the signatures. Initially, the backend requests the signature generated by the user’s message encrypted with their AuthKey and signed using the metamask plugin. After receiving the request, the backend validates the received signature.

Schiffman^32^ developed a DAuth authorization mechanism that permits users to access the services from distributed web applications in a specific and flexible manner. According to this system, DAuth oversees assigning and revoking protected resources by giving a policy-defined set of rules that eliminate the dependency on a centralized system.

Abbas^33^ reported an effective decentralized authentication system using blockchain to reduce the overhead communication latency of patient healthcare records in interconnected healthcare systems. This decentralized blockchain network helps to migrate patients and staff from one hospital to another without re-authentication. According to this system, when a patient submits a transaction in the hospital, the nursing station acts as a validator in an affiliated hospital, performs preliminary checks, such as signature verification and sufficient balances, and executes the transaction. After a successful transaction, the nursing station adds it to the ledger.

Suresh Babu^34^ proposed a distributed identity-based authentication scheme to provide trust within the resource-constrained IoT devices by delivering data protection and access control during unsecured communication. This model solves the single-point failure of public-key infrastructure (PKI) and private key generator (PKG) along with its key escrow problem.

Nagendra Kumar Nainar^35^ introduced a distributed authentication and validation system for user information, including data related to public keys within the blockchain. In this process, an electronic device produces a chunk of data, attaches the signature to the chunk of data, and transmits this chunk to one or more client devices in response to individual requests or the network address specified within the request. These signatures are produced by employing a private key of the electronic device. The electronic device stores the data, including details of a public key related to the private key, in a first ledger entry of a blockchain.

Balaji Balaraman^36^ presented the idea of a single sign-on solution using blockchain. In this case, suppose a system receives a registration request from the service provider, then the system conjures the smart contract to approve whether the credentials match a stored credential in the blockchain. Based on the login credential, the system creates a single sign-on token in response to the matching stored credential. The system transmits the single sign-on token to the client’s device and grants access to the system within the peer-to-peer network.

Vinit Kumar^37^ has proposed a Decentralized Open Authorization Framework in which the authorization server is split into two servers. Each server receives unique credentials and creates a unique access token. The individual access tokens are verified and combined into one token at the resource server. The resource server validates it, and grants access to the protected resources.

Padma^38^ has presented an authentication and authorization D-Auth mechanism for accessing serverless cloud applications by providing server-based OTP and token authentication. This mechanism uses a token Introspector to authorize users to request access services present in the serverless cloud.

**Table 4:** Summary of Notations.

Description	Notation
User	*U*
Identity provider—Ethereum	$IdP_{Eth}$
Identity provider—Hyperledger Fabric	$IdP_{HLF}$
Service provider	$S_{p}$
Web application	$W_{App}$
Decentralized identity	*DID*
Hash of DID	*Hash*(*DID*)
Secret sharing generate	$SSH_{Generate}$
Decentralized identity of mandatory share	$DID_{MS}$
Decentralized identity of second share	$DID_{S2}$
Decentralized identity of third share	$DID_{S3}$
Decentralized identity of fourth share	$DID_{S4}$
Hash of DID mandatory share	$Hash(DID_{MS})$
IPFS hash of DID second share	$IPFS_{Hash}(DID_{S2})$
IPFS hash of DID third share	$IPFS_{Hash}(DID_{S3})$
IPFS hash of DID fourth share	$IPFS_{Hash}(DID_{S4})$
Secret sharing combine	$SSH_{Combine}$
One of the given share	*OneOf*
Alternative share to OneOf function	*Other*
Adversary	$\mathscr {A}$
Ideal simulator	$\mathscr {S}_\mathscr {A}$
Probabilistic polynomial time	PPT
Random nonce	$\omega$

## Our proposed scheme

**Figure 4:**
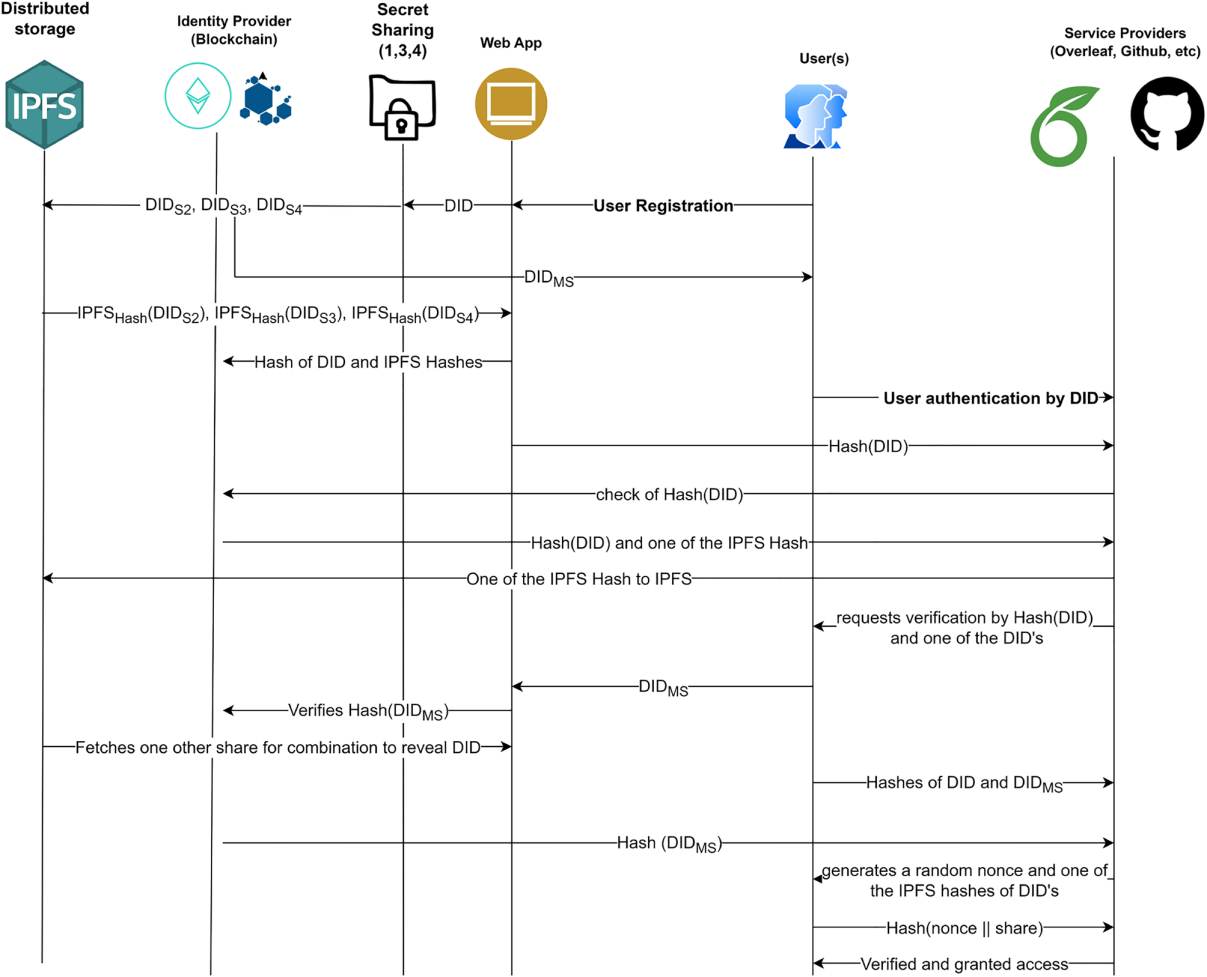
Proposed scheme.

Table 4 summarizes the notations used in this paper. As shown in Fig. 4, there are two main modules in our scheme. The first one is Identity Creation and Registration Phase and the second one is Identity Authentication Phase.

### Protocol design

Let us assume that user *U* wants to login to a service provide $S_{p}$ using the blockchain system [Ethereum ($IdP_{Eth}$) or Hyperledger Fabric ($IdP_{HLF}$)]. There are two key phases in performing this.

#### Identity creation and registration phase

Initially, a valid identity is created for the user that complies with W3C DID standards. Then comes the user registration phase. The details are elaborated as follows.

1. *U* submits the details ($Name || Email\_ID || SSN || Blood\_Group || Birth\_Date || Phone\_Number$) to the $W_{App}$ for creation of the *DID* that complies with W3C DID standards.
2. The *DID* is then passed to a (1,3,4) scheme $$SSH_{generate}$$ to generate four shares ($$DID_{MS}, DID_{S2}, DID_{S3}, DID_{S4})$$ as per Algorithm 1, out of which the first share is mandatory to regenerate the *DID*.
3. The $DID_{MS}$ is the important share that could reveal the DID on combining this with two of the remaining three shares. This should be kept private and secure by the *U*.
4. The three shares $DID_{S2}, DID_{S3}, DID_{S4}$ are stored in the *IPFS*. These shares can now be accessed with their hash values $IPFS_{Hash}(DID_{S2}), IPFS_{Hash}(DID_{S3}), IPFS_{Hash}(DID_{S4})$.
5. Finally, $(Hash(DID) || Hash(DID_{MS}) || IPFS_{Hash}(DID_{S2}) || IPFS_{Hash}(DID_{S3}) || IPFS_{Hash}(DID_{S4}))$ are submitted to the Blockchain through the smart contract/chaincode by the $W_{App}$.

#### Identity authentication phase

In this phase, the User *U* shall use the Decentralized Identity (*DID*) to perform single sign-on to the Service Provider ($S_{p}$).

1. *U* visits the $S_{p}$’s $W_{App}$ and sign-in either using $IdP_{Eth}$ or $IdP_{HLF}$ and provides the *DID*.
2. The $W_{App}$ calculates the *Hash*(*DID*) and sent to the $S_{p}$.
3. $S_{p}$ now verifies the *Hash*(*DID*) from the Blockchain to confirm the existence of the valid user.
4. If the provided *DID* belongs to the valid *U*, then ($Hash(DID) || OneOf(IPFS_{Hash}(DID_{S2}), IPFS_{Hash}(DID_{S3}), IPFS_{Hash}(DID_{S4})))$ is provided by the Blcockchain to the $S_{p}$.
5. $S_{p}$ now uses $OneOf(IPFS_{Hash}(DID_{S2}), IPFS_{Hash}(DID_{S3}), IPFS_{Hash}(DID_{S4}))$ to fetch one of the shares from the IPFS.
6. $S_{p}$ request for verification from *U* by providing $(Hash(DID) || OneOf(DID_{S2}, DID_{S3}, DID_{S4}))$.
7. *U* now uses the $DID_{MS}$ similar to a private key or password to authenticate and submit the same. The $W_{App}$ calculates the $Hash(DID_{MS})$ to verify it from the Blockchain.
8. If the hash is found matching, then the $W_{App}$ fetches one other share from IPFS and performs a combination operation, $SSH_{Combine}(DID_{MS}, OneOf(DID_{S2}, DID_{S3}, DID_{S4}), Other(DID_{S2}, DID_{S3}, DID_{S4}))$ to reveal the *DID* as given in Algorithm 2.
9. The *U* now shares the $(Hash_{Calculated}(DID) || Hash(DID_{MS}))$ to the $S_{p}$.
10. $S_{p}$ now verifies the $Hash(DID_{MS})$ from the Blockchain and verifies $Hash_{Calculated}(DID)$ by *U* is same as *Hash*(*DID*) it got initially, thus successfully verifying the *U*.
11. The $S_{p}$ will now generate a random nonce ($\omega$) and send $(\omega || OneOf(IPFS_{Hash}(DID_{S2}), IPFS_{Hash}(DID_{S3}), IPFS_{Hash}(DID_{S4}))$ to the *U*.
12. The *U* now uses $OneOf(IPFS_{Hash}(DID_{S2}), IPFS_{Hash}(DID_{S3}), IPFS_{Hash}(DID_{S4}))$, to fetch one of the shares from the IPFS as given by $S_{p}$, and $Hash(\omega + OneOf(Share))$ is computed. This computed hash value ($Hash_{Nonce+Share}$) is returned to the $S_{p}$.
13. $S_{p}$ now verifies ($Hash_{Nonce+Share}$), thus providing multifactor verification.

In the Identity Creation and Registration Phase, we uses smart contract (resp. chaincode) to store the hashes of the shares to the Ethereum (resp. Hyperledger Fabric) Blockchain. In the Identity Authentication Phase, the Web Application retrieves the shares from the Blockchain using the smart contract or chaincode. The share generation and secret reconstruction (i.e., Algorithm 1 and 2) are offchain computations. In our proposed model, we have used the Solidity programming to write the smart contract and deploy the application in Ethereum Ropsten Test Network Permissionless Blockchain. We have deployed the chaincode written in Go Language for Hyperledger Fabric Permissioned Blockchain. Web3.js was used to interface the User Interface with the Blockchain smart contracts.

### Key algorithm: secret sharing scheme

A Secret Sharing Scheme (SSS) is a cryptographic method for breaking a secret into multiple shares and distributing it among the participants. The dealer distributes the secret to the *n* participants as shares; when the required condition is fulfilled (a group of *t* participants which is a set in the qualified set - $\Gamma _\textit{Qual}$ joined), the secret can be reconstructed from the shares. This system is called (*t*, *n*)-secret sharing scheme. Here^39^, the least number of shares *t*, called a threshold, should be required to reconstruct the secret. An Adversary who discovers shares less than the threshold will not be able to get the secured secret. Blakley^40^ utilized a geometric approach to share the secret among the participants. According to this method, the secret key is the point in the *t-* dimensional space at which all the hyperplanes will intersect. Secret sharing schemes are beneficial for storing highly sensitive data, encryption keys, and missile launch codes. By distributing the data, among the participants, every individual has command and control over the data, thus minimizing the loss of data due to a single point of failure.

We use an ideal (1, *t*, *n*)-SSS to implement our framework. Let the set of participants is denoted as *P* = {$\textit{p}_1, \textit{p}_2, \textit{p}_3,\ldots , \textit{p}_\textit{n}$}. A SSS with minimal qualified set $\Gamma _\textit{QM}$ = {*A* $\in$ $\Gamma _\textit{Qual}$: $\textit{p}_1$ $\in$ *A* and $|\textit{A}|$=*t*} with $\textit{p}_1$ as the essential participant is called (1, *t*, *n*)-SSS. Arumugam *et al.*^41^ in 2014 proposed the strong access structure-based (1, *t*, *n*)-SSS, which is a special case of Ateniese *et al.*^42^ construction. For reconstructing the exact secret without any change, Cimato *et al.*^43^ in 2004, developed an ideal SSS using both OR and NOT as reconstruction operations. In this paper, we used the ideal (1, *t*, *n*)-SSS constructions^44^ developed by Praveen *et al.* in 2017. We demonstrated our experiments for (1, 3, 4)-SSS.
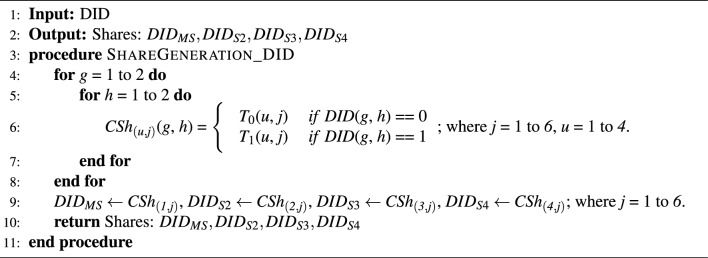


The following shows an example of (1, 3, 4)-SSS for sharing 0 and 1 bit. Let *P* $= \{\textit{p}_{1}, \textit{p}_{2}, \textit{p}_{3}, \textit{p}_{4}\}$ be the set of participants. The basis matrices $\textit{T}_{0} ($resp.$\textit{T}_{1})$ used for sharing bit 0 (resp. 1) are given as $$\textit{T}_{0} = \left[ \begin{array}{cccccc} 0 &{} 0 &{} 0 &{} 1 &{} 1 &{} 1\\ 0 &{} 1 &{} 1 &{} 1 &{} 0 &{} 1\\ 0 &{} 1 &{} 1 &{} 1 &{} 1 &{} 0 \\ 0 &{} 1 &{} 1 &{} 0 &{} 1 &{} 1 \\ \end{array}\right]$$ and $$\textit{T}_{1} = \left[ \begin{array}{cccccc} 0 &{} 0 &{} 0 &{} 1 &{} 1 &{} 1\\ 1 &{} 0 &{} 1 &{} 0 &{} 1 &{} 1\\ 1 &{} 1 &{} 0 &{} 0 &{} 1 &{} 1 \\ 0 &{} 1 &{} 1 &{} 0 &{} 1 &{} 1 \\ \end{array}\right]$$. Let the data (eg: DID) which we are going to share is represented as a matrix *DID* $$= \left[ \begin{array}{cc} 1 &{} 0 \\ 0 &{} 1 \\ \end{array}\right]$$. Any column permutation of the matrix $\textit{T}_{0} ($resp. $\textit{T}_{1})$ can be used for constructing shares for bit 0 (resp. 1). The minimal qualified set for (1, 3, 4)-SSS is $\Gamma _\textit{QM} = \{\{\textit{p}_{1},\textit{p}_{2},\textit{p}_{3}\}, \{\textit{p}_{1},\textit{p}_{2},\textit{p}_{4}\}, \{\textit{p}_{1},\textit{p}_{3},\textit{p}_{4}\}, \{\textit{p}_{1}, \textit{p}_{2}, \textit{p}_{3},\textit{p}_{4}\}\}$. For $\Gamma _\textit{QM}$ , six shares of each participant as generated using Algorithm 1 are given as follows.

$DID_{MS}$, i.e mandatory shares $$\textit{CSh}_{(1, 1)} = \textit{CSh}_{(1, 2)} = \textit{CSh}_{(1, 3)} = \left[ \begin{array}{cc} 0 &{} 0 \\ 0 &{} 0 \\ \end{array}\right]$$, $$\textit{CSh}_{(1, 4)} = \textit{CSh}_{(1, 5)} = \textit{CSh}_{(1, 6)} = \left[ \begin{array}{cc} 1 &{} 1 \\ 1 &{} 1 \\ \end{array}\right]$$ are distributed to $\textit{p}_1$.

$DID_{S2}$, i.e shares $$\textit{CSh}_{(2, 1)} = \left[ \begin{array}{cc} 1 &{} 0 \\ 0 &{} 1 \\ \end{array}\right]$$, $$\textit{CSh}_{(2, 2)} = \left[ \begin{array}{cc} 0 &{} 1 \\ 1 &{} 0 \\ \end{array}\right]$$, $$\textit{CSh}_{(2, 3)} = \left[ \begin{array}{cc} 1 &{} 1 \\ 1 &{} 1 \\ \end{array}\right]$$, $$\textit{CSh}_{(2, 4)} = \left[ \begin{array}{cc} 0 &{} 1 \\ 1 &{} 0 \\ \end{array}\right]$$, $$\textit{CSh}_{(2, 5)} = \left[ \begin{array}{cc} 1 &{} 0 \\ 0 &{} 1 \\ \end{array}\right]$$ and $$\textit{CSh}_{(2, 6)} = \left[ \begin{array}{cc} 1 &{} 1 \\ 1 &{} 1 \\ \end{array}\right]$$ are distributed to $\textit{p}_2$.

$DID_{S3}$, i.e shares $$\textit{CSh}_{(3, 1)} = \left[ \begin{array}{cc} 1 &{} 0 \\ 0 &{} 1 \\ \end{array}\right]$$, $$\textit{CSh}_{(3, 2)} = \left[ \begin{array}{cc} 1 &{} 1 \\ 1 &{} 1 \\ \end{array}\right]$$, $$\textit{CSh}_{(3, 3)} = \left[ \begin{array}{cc} 0 &{} 1 \\ 1 &{} 0 \\ \end{array}\right]$$, $$\textit{CSh}_{(3, 4)} = \left[ \begin{array}{cc} 0 &{} 1 \\ 1 &{} 0 \\ \end{array}\right]$$, $$\textit{CSh}_{(3, 5)} = \left[ \begin{array}{cc} 1 &{} 1 \\ 1 &{} 1 \\ \end{array}\right]$$ and $$\textit{CSh}_{(3, 6)} = \left[ \begin{array}{cc} 1 &{} 0 \\ 0 &{} 1 \\ \end{array}\right]$$ are distributed to $\textit{p}_3$.

$DID_{S4}$, i.e shares $$\textit{CSh}_{(4, 1)} = \left[ \begin{array}{cc} 0 &{} 0 \\ 0 &{} 0 \\ \end{array}\right]$$, $$\textit{CSh}_{(4, 2)} = \left[ \begin{array}{cc} 1 &{} 1 \\ 1 &{} 1 \\ \end{array}\right]$$, $$\textit{CSh}_{(4, 3)} = \left[ \begin{array}{cc} 1 &{} 1 \\ 1 &{} 1 \\ \end{array}\right]$$, $$\textit{CSh}_{(4, 4)} = \left[ \begin{array}{cc} 0 &{} 0 \\ 0 &{} 0 \\ \end{array}\right]$$, $$\textit{CSh}_{(4, 5)} = \left[ \begin{array}{cc} 1 &{} 1 \\ 1 &{} 1 \\ \end{array}\right]$$ and $$\textit{CSh}_{(4, 6)} = \left[ \begin{array}{cc} 1 &{} 1 \\ 1 &{} 1 \\ \end{array}\right]$$ are distributed to $\textit{p}_4$.
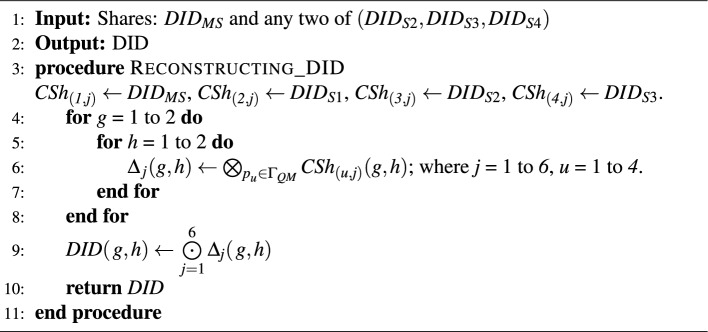


The below-given procedure as per Algorithm 2 is used to reconstruct *DID*. Let us denote $\bigotimes$ as Boolean OR operation and $\bigodot$ as Boolean AND operation. According to SSS, participants in any one of the qualified sets of $\Gamma _\textit{QM}$ can reconstruct a secret. So here in this example, the qualified set we selected is $\{\textit{p}_{1},\textit{p}_{2},\textit{p}_{3}\}$ in the $\Gamma _\textit{QM}$. So reconstruction of *DID* using the shares of the participants $\textit{p}_\textit{1}$, $\textit{p}_\textit{2}$ and $\textit{p}_\textit{3}$ is given as follows. First, generate all $\Delta _{\textit{j}}$ using bit -by-bit XOR of participants shares, i.e $$\Delta _{1} = \bigotimes \limits _{u=1}^3\textit{CSh}_{(u, 1)}=\left[ \begin{array}{cc} 1 &{} 0 \\ 0 &{} 1 \\ \end{array}\right]$$, $$\Delta _{2} = \bigotimes \limits _{u=1}^3\textit{CSh}_{(u, 2)}= \left[ \begin{array}{cc} 1 &{} 1 \\ 1 &{} 1 \\ \end{array}\right]$$, $$\Delta _{3} = \bigotimes \limits _{u=1}^3\textit{CSh}_{(u, 3)}= \left[ \begin{array}{cc} 1 &{} 1 \\ 1 &{} 1 \\ \end{array}\right]$$, $$\Delta _{4} = \bigotimes \limits _{u=1}^3\textit{CSh}_{(u, 4)}= \left[ \begin{array}{cc} 1 &{} 1 \\ 1 &{} 1 \\ \end{array}\right]$$, $$\Delta _{5} = \bigotimes \limits _{u=1}^3\textit{CSh}_{(u, 5)}= \left[ \begin{array}{cc} 1 &{} 1 \\ 1 &{} 1 \\ \end{array}\right]$$ and $$\Delta _{6} = \bigotimes \limits _{u=1}^3\textit{CSh}_{(u, 6)}= \left[ \begin{array}{cc} 1 &{} 1 \\ 1 &{} 1 \\ \end{array}\right]$$. Now the DID is obtained by applying bit-by-bit AND operation of all $\Delta _{\textit{j}}$, i.e $$DID = \bigodot \limits _{j=1}^6\Delta _{\textit{j}}=\left[ \begin{array}{cc} 1 &{} 0 \\ 0 &{} 1 \\ \end{array}\right]$$.

## Security considerations

### Informal security analysis

1. *Decentralization and Immutable Ledger:* Using a blockchain system introduces decentralization and an immutable ledger, which can enhance security. Since the user identity information is distributed across the blockchain network, it becomes more resilient against single points of failure and tampering.
2. *Privacy and Confidentiality:* The secret sharing scheme, where the user’s identity is split into multiple shares stored in IPFS, can improve privacy and confidentiality. It ensures that no single entity holds complete information about the user’s identity, reducing the risk of data breaches. Also, our assumption is all communications in our protocol are encrypted.
3. *Data Integrity:* The immutability of the blockchain ensures that once the user identity is recorded, it cannot be altered or deleted without consensus from the network. This prevents unauthorized changes to user data, enhancing data integrity.
4. *Secure Hashing:* Cryptographic hashing for storing and verifying user information adds an extra layer of security. Hashing ensures that sensitive information, like the user’s DID and shares, is not stored in plaintext, making it difficult for attackers to retrieve the original data.
5. *Authentication Strength:* The combination of the secret sharing scheme and blockchain-based verification for authentication may provide robust security, especially if the secret shares are generated and stored securely.

### Universal composability security framework

In this section, we shall analyze the security of the proposed solution under the universal composability security framework.The basic objective of the Universal Composability (UC) framework is to guarantee that any key exchange protocol provides the same security as any other protocol which wants to set up session keys between two parties, even when it runs in parallel with an arbitrary set of other protocols in a distributed communication network. Here we use UC Framework to authenticate the Decentralized Identifier, and our assumption is that all communication in our protocol is encrypted.

UC framework follows the approach of “security by emulation of an ideal process.”^45, 46^ That means a real protocol $\pi _r$ realizes the task *T*, if there is an adversary $\mathscr {A}$ attacks $\pi _r$, there also exists a simulator $\mathscr {S}_\mathscr {A}$ that can do an Adversary Simulation by interacting with Ideal Process $\mathscr {F}$. Also, proof of indistinguishability means that no environment ($\mathscr {Z}$) can conclude with a non-negligible probability of success whether it is interacting with $\pi _{r}$ and $\mathscr {A}$ or with $\mathscr {F}$ and $\mathscr {S}_\mathscr {A}$ for *T*. In our protocol, the task *T* is the SSO-based authentication of the Decentralized Identifier. The Ideal Processes in our scheme are Secret Sharing or Secret Reconstruction ($\mathscr {F}_{\text {SS}}$), $W_{App}$ Operations ($\mathscr {F}_{\text {WApp}}$), IPFS Operations ($\mathscr {F}_{\text {IPFS}}$)and Blockchain Operations($\mathscr {F}_{\text {BO}}$).

### Analysis of proposed scheme

Our assumption is all communication is encrypted and transferred via the Internet (HTTPS). Let us assume that user *U* wants to login to a service provide $S_{p}$ using the blockchain system *IdP*[Ethereum ($IdP_{Eth}$) or Hyperledger Fabric ($IdP_{HLF}$)]. There are two key phases in performing this.

#### Identity creation phase

The user must create a valid identity using the selected blockchain system’s supported wallet or Certificate Authority (*CA*).

1. *U* submits the details *CT*=($Name || Email\_ID || SSN || Blood\_Group || Birth\_Date || Phone\_Number$) to the *IdP* through the $W_{App}$ for creation of the $U_{I} (DID)$ that complies with W3C DID standards. In UC this communication is represented as
  *U* sends (Register, reg, *CT*, *U*, $W_{App}$) to $W_{App}$, where *reg* is the registration tag and $\mathscr {S}_\mathscr {A}$. $\mathscr {S}_\mathscr {A}$ now sends (ask, reg, *CT*, *U*, $W_{App}$) to $W_{App}$.
  $W_{App}$ sends (GenerateDID, reg, *DID*, $W_{App}$, *U*) to $\mathscr {F}_{\text {WApp}}$. $\mathscr {F}_{\text {WApp}}$ generate *DID* then transfer (res, reg, *DID*, $W_{App}$, *U*) to *U* and $\mathscr {S}_\mathscr {A}$.$\mathscr {S}_\mathscr {A}$ now sends (res, reg, *DID*, $W_{App}$, *U*) to *U*.
  $W_{App}$ sends (Submit, reg, *CT*, $W_{App}$, *IdP*) to $\mathscr {F}_{\text {BO}}$ and $\mathscr {S}_\mathscr {A}$.
2. The *DID* is then passed to a $$SSH(1,3,4)_{generate}$$ to generate four shares ($$DID_{MS}, DID_{S2}, DID_{S3}, DID_{S4})$$, out of which $$DID_{MS}$$ is mandatory to regenerate the *DID* and this should be kept private and secure by the *U*. The three shares $$DID_{S2}, DID_{S3}, DID_{S4}$$ are stored in the *IPFS* by *U*. Shares can now be accessed with their hash values $$IPFS_{Hash}(DID_{S2}),IPFS_{Hash}(DID_{S3}), IPFS_{Hash}(DID_{S4})$$. In UC this communication is represented as
  After receiving (res, reg, *DID*, $W_{App}$, *U*), *U* sends (SecretShare, reg, *DID*, *U*) to $\mathscr {F}_{\text {SS}}$. $\mathscr {F}_{\text {SS}}$ create shares and writes down (store, $DID_{MS}$, *U*).
  *U* sends (ask, store, reg, $DID_{S2}$, *U*, *IPFS*) to $\mathscr {F}_{\text {IPFS}}$ and $\mathscr {S}_\mathscr {A}$. Now $\mathscr {S}_\mathscr {A}$ sends (ask, store, reg, $DID_{S2}$, *U*, *IPFS*) to $\mathscr {F}_{\text {IPFS}}$. $\mathscr {F}_{\text {IPFS}}$ generates $IPFS_{Hash}(DID_{S2})$ and sends (res, reg, $IPFS_{Hash}(DID_{S2})$, *IPFS*, *U*) to *U* and $\mathscr {S}_\mathscr {A}$. Now $\mathscr {S}_\mathscr {A}$ sends (res, reg, $IPFS_{Hash}(DID_{S2})$, *IPFS*, *U*) to *U*.
  Same above given adversarial simulation will happen while submitting shares $DID_{S3}$ and $DID_{S4}$ to *IPFS* by *U*.
3. Finally, *CT*=$(Hash(DID) || Hash(DID_{MS}) || IPFS_{Hash}(DID_{S2}) || IPFS_{Hash}(DID_{S3}) || IPFS_{Hash}(DID_{S4}))$ are submitted to the $\mathscr {F}_{BO}$ through the smart contract/chaincode by the $W_{App}$. In UC this communication is represented as
  *U* sends (submit, *CT*, *U*, $W_{App}$) to $W_{App}$ and $\mathscr {S}_\mathscr {A}$. $\mathscr {S}_\mathscr {A}$ now sends (submit, *CT*, reg, *U*, $W_{App}$) to $W_{App}$.
  $W_{App}$ sends (submit, reg, *CT*, $W_{App}$, *IdP*) to $\mathscr {F}_{\text {BO}}$ and $\mathscr {S}_\mathscr {A}$.

#### Identity authentication phase

In this phase, the User *U* shall use the Decentralized Identity (*DID*) to perform single sign-on to the Service Provider ($S_{p}$).

1. *U* visits the $S_{p}$’s $W_{App}$ and sign-in either using *IdP*($IdP_{Eth}$ or $IdP_{HLF}$) and provides the *DID*. The $W_{App}$ calculates the *Hash*(*DID*) and sent to the $S_{p}$. $S_{p}$ verifies the *Hash*(*DID*) from the *IdP* to confirm the existence of the valid *U*. If the provided *DID* belongs to the valid *U*, then *CT*=($Hash(DID) || OneOf(IPFS_{Hash}(DID_{S2}), IPFS_{Hash}(DID_{S3}), IPFS_{Hash}(DID_{S4})))$ is provided by the *IdP* to the $S_{p}$. In UC this communication is represented as
  *U* sends (Sign-in, auth, *DID*, *U*, $W_{App}$) to $W_{App}$ and $\mathscr {S}_\mathscr {A}$ , where *auth* is the authentication tag. $\mathscr {S}_\mathscr {A}$ now sends (auth, *DID*, *U*, $W_{App}$) to $W_{App}$.
  $W_{App}$ sends (GenerateHashDID, auth, *DID*, $W_{App}$, $S_{p}$) to $\mathscr {F}_{\text {WApp}}$. $\mathscr {F}_{\text {WApp}}$ then generate *Hash*(*DID*) and transfer (auth, *Hash*(*DID*), $W_{App}$, $S_{p}$) to $S_{p}$ and $\mathscr {S}_\mathscr {A}$.$\mathscr {S}_\mathscr {A}$ now sends (auth, *Hash*(*DID*), $W_{App}$, $S_{p}$) to $S_{p}$.
  $S_{p}$ sends (Checking, auth, *Hash*(*DID*), $S_{p}$, *IdP*) to $\mathscr {F}_{\text {BO}}$ and $\mathscr {S}_\mathscr {A}$.
  If the provided *DID* belongs to the valid *U*, then $\mathscr {F}_{\text {BO}}$ will transfer (auth, *CT*, *IdP*, $S_{p}$) to $S_{p}$ and $\mathscr {S}_\mathscr {A}$.
2. $S_{p}$ now uses *CT*=$OneOf(IPFS_{Hash}(DID_{S2}), IPFS_{Hash}(DID_{S3}), IPFS_{Hash}(DID_{S4}))$ to fetch one of the shares from the IPFS. In UC this communication is represented as, $S_{p}$ sends (ask, auth, *CT*, $S_{p}$, *IPFS*) to $\mathscr {F}_{\text {IPFS}}$ and $\mathscr {S}_\mathscr {A}$. Now $\mathscr {S}_\mathscr {A}$ sends (ask, auth, *CT*, $S_{p}$, *IPFS*) to $\mathscr {F}_{\text {IPFS}}$.
3. IPFS will send *CT*=$OneOf(DID_{S2}, DID_{S3}, DID_{S4})$ to $S_{p}$. In UC this communication is represented as, $\mathscr {F}_{\text {IPFS}}$ sends (res, auth, *CT*, *IPFS*, $S_{p}$) to $S_{p}$ and $\mathscr {S}_\mathscr {A}$. Now $\mathscr {S}_\mathscr {A}$ sends (res, auth, *CT*, *IPFS*, $S_{p}$) to $S_{p}$.
4. $S_{p}$ request for verification from *U* by providing *CT*=$(Hash(DID) || OneOf(DID_{S2}, DID_{S3}, DID_{S4}))$.In UC this communication is represented as, $S_{p}$ sends (ask, auth, *CT*, $S_{p}$, *U*) to *U* and $\mathscr {S}_\mathscr {A}$. Now $\mathscr {S}_\mathscr {A}$ sends (ask, auth, *CT*, $S_{p}$, *U*) to *U*.
5. *U* now uses the $DID_{MS}$ similar to a private key or password to authenticate and submit the same. The $W_{App}$ calculates the $Hash(DID_{MS})$ to verify it from the $\mathscr {F}_{BO}$. If the hash is found matching, then the $W_{App}$ fetches one other share from IPFS and performs, $SSH_{Combine}(DID_{MS}, OneOf(DID_{S2}, DID_{S3}, DID_{S4}), Other(DID_{S2}, DID_{S3}, DID_{S4}))$ to reveal the *DID*. $SSH_{Combine}$ is implemented by the ideal process $\mathscr {F}_{\text {SS}}$ to generate the *DID*.
6. *U* now shares the *CT*=$(Hash_{Calculated}(DID) || Hash(DID_{MS}))$ to the $S_{p}$. $S_{p}$ now verifies the $Hash(DID_{MS})$ from the *IdP* and verifies $Hash_{Calculated}(DID)$ by *U* is same as *Hash*(*DID*) it got initally, thus successfully verifying the *U*. In UC this communication is represented as, *U* sends (Verify, auth, *CT*, *U*, $S_{p}$) to $S_{p}$ and $\mathscr {S}_\mathscr {A}$. $\mathscr {S}_\mathscr {A}$ sends (Verify, auth,*CT*, *U*, $S_{p}$) to $S_{p}$. $S_{p}$ sends (Check, auth, $Hash(DID_{MS})$, $S_{p}$, *IdP*) to $\mathscr {F}_{\text {BO}}$ and $\mathscr {S}_\mathscr {A}$. $S_{p}$ also sends (Check, auth, $Hash_{Calculated}(DID)$, $S_{p}$, *IdP*) to $\mathscr {F}_{\text {BO}}$ and $\mathscr {S}_\mathscr {A}$.
7. The $S_{p}$ will now generate a random nonce ($\omega$) and send *CT*=$(\omega || OneOf(IPFS_{Hash}(DID_{S2}), IPFS_{Hash}(DID_{S3}), IPFS_{Hash}(DID_{S4}))$ to the *U*. In UC this communication is represented as, $S_{p}$ sends (Multifactor, auth, *CT*, $S_{p}$, *U*) to *U* and $\mathscr {S}_\mathscr {A}$.
8. The *U* now uses $OneOf(IPFS_{Hash}(DID_{S2}), IPFS_{Hash}(DID_{S3}), IPFS_{Hash}(DID_{S4}))$, to fetch one of the shares from the IPFS as given by $S_{p}$, and *CT*=$Hash(\omega + OneOf(Share))$ is computed. This computed hash value is returned to the $S_{p}$. In UC this communication is represented as, *U* sends (Multifactor, auth, *CT*, *U*, $S_{p}$) to $S_{p}$ and $\mathscr {S}_\mathscr {A}$.
9. $S_{p}$ now verifies this Hash, thus providing multifactor verification.

#### Security against attack scenarios

Next, we define an event $E$ that $\mathscr {S}_\mathscr {A}$ can modify the communications in the Identity creation phase and Identity authentication phase by calculating new tags like $\textit{reg}_{a}$ and $\textit{auth}_{a}$ respectively instead of *reg* and *auth*. $\mathscr {S}_\mathscr {A}$ can also forge the *CT* value with a new $\textit{CT}_{a}$. The calculation of these values includes hash functions or random functions, and since it is challenging to construct a PPT algorithm to find a collision of hash functions or random functions, the success rate of event $E$ is negligible^46^.

## Performance analysis

**Figure 5:**
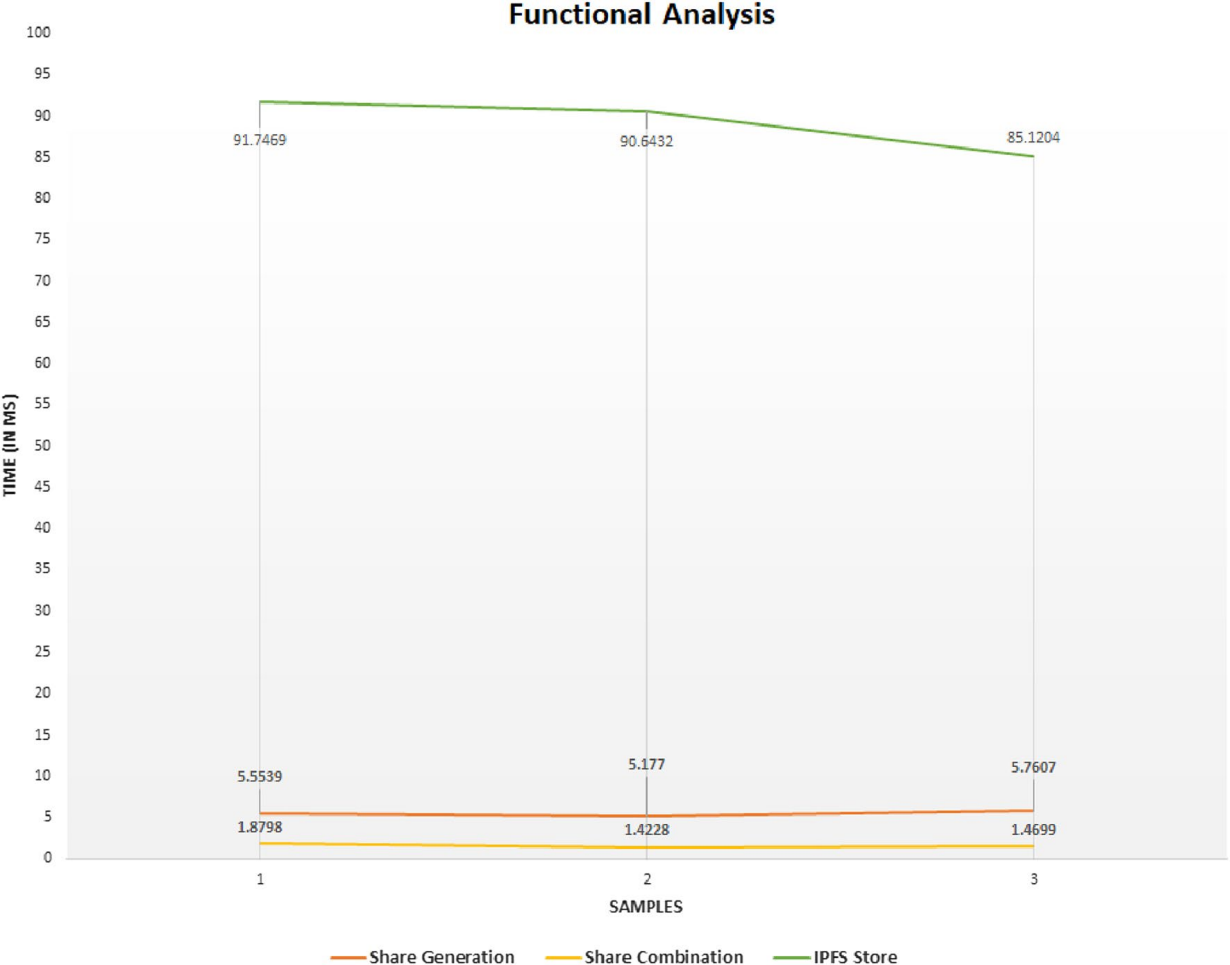
Analysis of functional time.

The performance of the proposed solution depends on the performance of the blockchain (Ethereum or Hyperledger Fabric), IPFS, and the secret sharing scheme. We have used a Test network in the case of Ethereum Blockchain, but in real-time, we will use Ethereum Mainnet, where the performance might differ. The transaction time depends on the network load and the gas fees paid. In IPFS, the storage and retrieval time depends on network latency. With the above-said considerations, the average time to generate shares from the *DID* is $\approx 4$–6 ms; uploading a share to IPFS is $\approx 25$–28 ms, and the average time to add this identity information to the blockchain can be considered as $\delta$. Therefore the average time for identity generation and storing the identity in the blockchain takes $\approx 30$–$36 \textrm{ms} + \delta s$, where delta is the time required to store the transaction in the blockchain. Similarly, for the authentication, the average time for share combination is $\approx 1.6 ms$ as shown in Fig. 5.

## Discussion

This section discusses the limitations of the previous works and how our solution surpasses these challenges as shown in Table 5. We also discuss the limitations of our work.

Soumyashree^29^ have implemented the authentication mechanism using permissionless blockchain only. In our paper, we implemented the scheme for the permissioned and permissionless blockchains. This dual implementation, along with the Secret Sharing mechanism, has resulted in improving security. Hadjer Benhadj^30^ framework involves using keys for authentication, which requires more computational power. On the other hand, we are using a secret sharing mechanism where users can authenticate independently without using any trusted third party. The issue with Schiffman^32^ framework is that the DAuth works on policy-defined rules that are not universally compatible with most *IdP*s. In our framework, there is no inclusion of rules; hence, it has the flexibility to work with any *IdP*. In the case of decentralized frameworks presented by Kumar V.^37^ and Padma P.^38^ the authorization is done based on two third-party servers and enterprise private cloud respectively. In contrast, our framework stores uses the blockchain network which will be more trustworthy.

**Table 5:** Comparison of Existing work with SSH-DAuth.

Technology	Soumyashree^29^	Hadjer B.^30^	Schiffman^32^	Kumar^37^	Padma^38^	SSH-DAuth
Distributed	$\checkmark$	$\checkmark$	$\checkmark$	$\checkmark$	$\checkmark$	$\checkmark$
msBlockchain	$\checkmark$	$\checkmark$	$\checkmark$			$\checkmark$
DID						$\checkmark$
IPFS						$\checkmark$
Secret sharing						$\checkmark$

We have considered Ethereum Blockchain and Hyperledger Fabric for implementing our proposed solution. We have taken this experiment to show that our model is blockchain agnostic. The model should be implemented using the corresponding smart contract / chaincode language; otherwise, the model is the same irrespective of the blockchain. Below, we present the limitations of our proposed solutions as per our understanding and analysis. These limitations and implications could lead to further work and research.

1. *Mandatory Share Protection:* The security of the user’s DID relies heavily on the secrecy and protection of the $DID_{MS}$ share, acting as a private key. If this share is compromised, an attacker could gain unauthorized access to the user’s identity.
2. *Smart Contract Vulnerabilities:* The smart contract deployed on the blockchain must be thoroughly audited to avoid security vulnerabilities and withstand potential attacks.
3. *Centralization of Secret Shares:* While the secret sharing scheme distributes shares across IPFS, if all shares are stored in a centralized IPFS node or managed by a single entity, it could introduce a single point of failure and compromise security.
4. *Blockchain and IPFS Security:* The security of the chosen blockchain (Ethereum or Hyperledger Fabric) and IPFS infrastructure are critical. Attackers could exploit vulnerabilities in the blockchain protocol or IPFS implementation.
5. *Transaction Anonymity:* The proposed approach does not explicitly address transaction anonymity, and the linkage of a user’s DID to their actions on the blockchain might reduce user privacy.

## Conclusion and future works

This paper describes a secure and robust distributed multifactor authentication & authorization protocol using DIDs and secret sharing based on blockchain. The proposed work addresses several authentication issues, such as the role of intermediaries, insecure storage, and mutability, that occur in a traditional centralized system by leveraging blockchain technology. By incorporating the concepts of DID and Secret Sharing, our proposed solution improves the security, privacy, and trust of the entire ecosystem while allowing for the selective disclosure of sensitive information. The use of private IPFS and the encryption of its data add to the data’s security and limit the flow of information within the network. Based on our findings, we can conclude that adding DID and Secret Sharing increases trust, privacy, and scalability in a peer-to-peer application built on Ethereum. The results show that the proposed solution provides a seamless and faster user experience than a centralized repository authenticating system.

It might be possible to decentralize different types of SSOs like Enterprise SSO (ESSO), Cross-Domain SSO, and Federated SSO. These systems can also adopt a multi-factor authentication framework.

## Data Availability

The datasets used and/or analyzed during the current study are available from the corresponding author upon reasonable request. The project GitHub repository could be referred^47^.
